# MultiKano: an automatic cell type annotation tool for single-cell multi-omics data based on Kolmogorov–Arnold network and data augmentation

**DOI:** 10.1093/procel/pwae069

**Published:** 2024-12-10

**Authors:** Siyu Li, Xinhao Zhuang, Songbo Jia, Songming Tang, Liming Yan, Heyang Hua, Yuhang Jia, Xuelin Zhang, Yan Zhang, Qingzhu Yang, Shengquan Chen

**Affiliations:** School of Mathematical Sciences and LPMC, Nankai University, Tianjin 300071, China; Capital University of Physical Education and Sports, Beijing 100191, China; Capital University of Physical Education and Sports, Beijing 100191, China; School of Mathematical Sciences and LPMC, Nankai University, Tianjin 300071, China; School of Medicine, Tsinghua University, Beijing 100084, China; School of Mathematical Sciences and LPMC, Nankai University, Tianjin 300071, China; School of Mathematical Sciences and LPMC, Nankai University, Tianjin 300071, China; Capital University of Physical Education and Sports, Beijing 100191, China; Capital University of Physical Education and Sports, Beijing 100191, China; Capital University of Physical Education and Sports, Beijing 100191, China; School of Mathematical Sciences and LPMC, Nankai University, Tianjin 300071, China

**Dear Editor,**

The breakthrough in single-cell omics sequencing technologies has provided an unprecedented level of detail, allowing biologists to explore the patterns of gene activity, and the dynamics of cellular function at the resolution of individual cells. At the forefront of this revolution is single-cell RNA sequencing (scRNA-seq), which measures gene expression of individual cells to characterize transcriptional heterogeneity. Additionally, other single-cell assays, such as single-cell assay for transposase-accessible chromatin using sequencing (scATAC-seq), shed light on cellular heterogeneity at the epigenetic level, enhancing our understanding of transcriptional regulation. However, while single-omics sequencing techniques provide valuable insights, they may not capture the intricate relationships between biomolecules in single cells due to their restriction to only one type of omics data. To bridge this gap, recent advancements have led to the development of several joint profiling methods (Cao et al., 2018; Chen et al., 2019; Luecken et al., 2021; Ma et al., 2020), which enable the simultaneous measurement of gene expression and chromatin accessibility, offering a holistic view of the gene regulatory landscape in individual cells.

Nevertheless, the effective utilization of single-cell multi-omics data hinges critically on the accurate annotation of cell types, a prerequisite that supports further downstream analyses such as the precise identification of cell-type-specific regulatory elements and the construction of detailed gene regulatory networks. Consequently, cell type annotation is a core step in single-cell multi-omics data analysis. To identify cell populations in single-cell datasets, the common approach involves unsupervised clustering, followed by manually assigning cell type labels to each cluster according to the prior knowledge (Cao et al., 2019). However, the cluster-based annotation methods assume that all cells within a cluster belong to the same cell type. This assumption frequently fails, as one cluster commonly comprises minor populations of various cell types in addition to a major cell type. Moreover, as the number of individual cells being profiled increases exponentially, the manual annotation method becomes labor-intensive, difficult to reproduce, and time-consuming.

A more efficient and accurate alternative is automatic cell type annotation, which leverages well-labeled datasets to train models and then applies the trained model to annotate newly generated datasets. Many computational methods specifically designed for scRNA-seq data have been proposed. For example, an automatic cell type annotation method to classify single cells based on singular value decomposition and a support vector machine (SVM) model, namely scPred, has been proven superior in several studies (Alquicira-Hernandez et al., 2019). Moreover, Chen et al. recently proposed a multi-head self-attention network called TOSICA for interpretable cell type annotation in scRNA-seq data Chen et al., (2023). Additionally, there are also several computational methods tailored to scATAC-seq data. EpiAnno is the first automatic cell type annotation method specifically designed for scATAC-seq data, which utilizes a Bayesian neural network to perform annotation (Chen et al., 2022). More recently, Zeng et al. proposed SANGO, an accurate and scalable graph-based method for annotating cells within scATAC-seq data by integrating DNA sequence information (Zeng et al., 2024). Furthermore, conventional machine learning methods, such as SVM, Random Forest (RF), and Multi-Layer Perceptron (MLP), have demonstrated robust performance in exclusively annotating either scRNA-seq data or scATAC-seq data (Abdelaal et al., 2019; Chen et al., 2022; Ma et al., 2021). These single-omics annotation methods can be applied to cell type annotation tasks for single-cell multi-omics data, that is, utilizing one omics type such as scRNA-seq or scATAC-seq profiles to determine the cell type of each cell. However, the single-omics methods fail to fully harness the information from multi-omics profiles, restricting their ability to capture the complexity and diversity of cells. Consequently, there is an urgent need to develop an automatic cell type annotation method tailored to single-cell multi-omics data.

To fill these gaps, we proposed MultiKano, to our best knowledge, the first method that integrates single-cell transcriptomic and chromatin accessibility data for automatic cell type annotation. MultiKano introduces a novel data augmentation strategy based on paired scRNA-seq and scATAC-seq profiles and incorporates the advanced Kolmogorov–Arnold Network (KAN) (Liu et al., 2024) for enhancing the generalization capabilities of the model. The architecture of MultiKano is structured into three main modules: the data preprocessing module, the data augmentation module, and the KAN module (Fig. 1; Text S1). Specifically, for a given paired single-cell multi-omics dataset, MultiKano first performs data preprocessing on the scRNA-seq and scATAC-seq profiles separately (Text S1). To capture cell heterogeneity in the exceedingly noisy single-cell multi-omics data, we further design a data augmentation module. The fundamental principle behind this module is that two cells of the same cell type possess similar biological characteristics, thus enabling the different omics profiles of the two cells to match each other. Specifically, for two cells of the same type, the levels of gene expression and chromatin accessibility of the two cells are remarkably consistent. Leveraging this principle, we can create synthetic cells by matching the scRNA-seq profile of one cell with the scATAC-seq profile of another cell (Text S1). After obtaining synthetic cells, we concatenate the scRNA-seq and scATAC-seq profiles of each cell to serve as the input of the KAN model (Text S1). The KAN model, which is inspired by the Kolmogorov–Arnold representation theorem, has no linear weight matrices at all, instead, each weight parameter is replaced by a learnable 1D function parametrized as a spline. This configuration allows KAN to simply sum incoming signals linearly without applying any non-linearities. By employing learnable activation functions along the network’s edges, KAN achieves excellent flexibility and generalizability, enabling efficient learning of complex nonlinear mappings and reducing the risk of overfitting. Therefore, KAN is an algorithm well-suited for single-cell data analysis, as single-cell data often features high dimensionality and extreme sparsity. However, to our best knowledge, the KAN model has not been previously used for annotating single-cell data.

**Figure 1.**
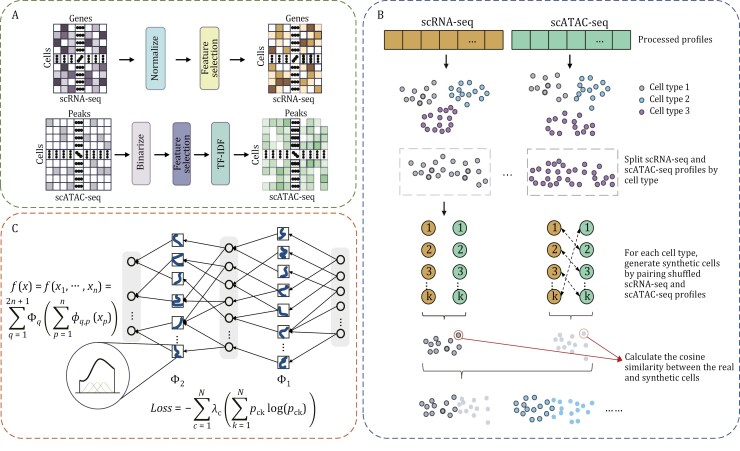
Overview of MultiKano. (A) Data preprocessing module: Given a paired single-cell multi-omics dataset, MultiKano first preprocesses the scRNA-seq and scATAC-seq profiles separately. (B) Data augmentation module: Using the preprocessed profiles, MultiKano generates synthetic cells by pairing the scRNA-seq profile of one cell with the scATAC-seq profile of another cell from the same cell type. (C) KAN module: Finally, MultiKano concatenates the two omics profiles of each cell and utilizes the KAN model to predict the cell type.

To evaluate whether MultiKano, which integrates single-cell transcriptomic and chromatin accessibility data, outperforms existing automatic cell type annotation methods tailored to single-omics data, we conducted five-fold cross-validation on six paired single-cell multi-omics datasets. The datasets include Cortex (Chen et al., 2019), Brain (Ma et al., 2020), SkinA (Ma et al., 2020), SkinB (Ma et al., 2020), Kidney (Cao et al., 2018), and PBMC (Luecken et al., 2021), which are profiled from different species, tissues, and protocols (Text S2; Table S1). As suggested in the recent benchmark studies (Abdelaal et al., 2019; Ma et al., 2021), we evaluated the annotation performance by using the metrics of Accuracy, Cohen’s kappa value (Kappa) and macro F1 score (F1-macro) (Text S3). Firstly, we compared MultiKano with cell type annotation methods only using scRNA-seq profiles. These methods include scPred (Alquicira-Hernandez et al., 2019), TOSICA (Chen et al., 2023), and three well-performed conventional machine learning methods, including SVM, RF, and MLP (Text S4). Additionally, we included a comparison with Random Guessing (RG) to illustrate the baseline performance and emphasize the effectiveness of MultiKano. RG refers to randomly selecting a cell type from the training set to predict the cell type for the test cell. We used the aforementioned six single-cell multi-omics datasets for evaluation, where MultiKano leveraged both types of omics profiles, while the baseline methods only utilized scRNA-seq profiles. Note that scPred encountered an error “line search fails” on the SkinA dataset. As shown in Figs. 2A and S1, MultiKano exhibited the best overall performance across all six datasets, with particularly pronounced advantages in the metrics of Accuracy and Kappa. scPred provided the second-best overall performance, aligning with the recent benchmark results (Abdelaal et al., 2019; Ma et al., 2021). Additionally, we conducted one-sided paired Wilcoxon signed-rank tests to determine whether MultiKano significantly outperformed scPred across these datasets. The results confirmed that MultiKano exceeded scPred across all metrics, with *P*-values of 2.980 × 10^−8^ for Accuracy, 2.980 × 10^−8^ for Kappa, and 0.237 for F1-macro.

**Figure 2.**
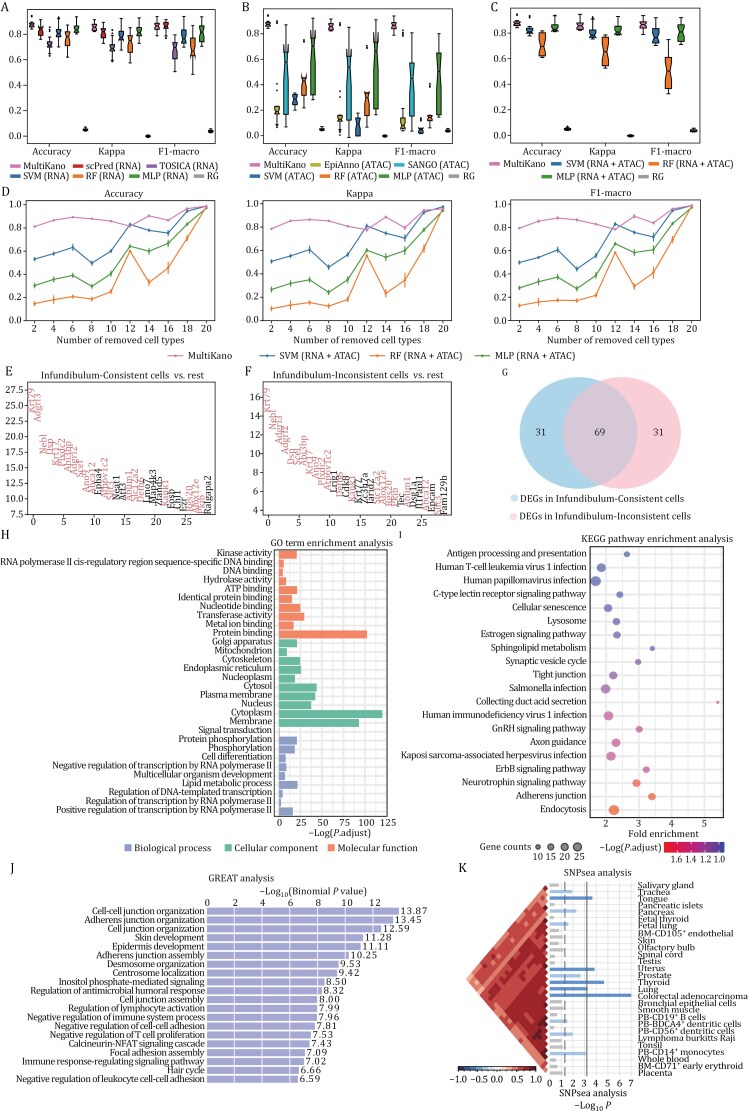
The performance of MultiKano and baseline methods. (A) The boxplot of MultiKano and baseline methods for scRNA-seq data as well as RG on the annotation performance across six single-cell multi-omics datasets. (B) The boxplot of MultiKano and baseline methods for scATAC-seq data as well as RG on the annotation performance across six single-cell multi-omics datasets. (C) The boxplot of MultiKano and conventional machine learning methods using multi-omics data as well as RG on the annotation performance across six single-cell multi-omics datasets. (D) The robustness of MultiKano and machine learning methods using multi-omics data to the number of cell types on the Cortex dataset. (E) The top 30 DEGs in Infundibulum-Consistent cells. (F) The top 30 DEGs in Infundibulum-Inconsistent cells. The overlapping genes are highlighted. (G) Venn diagram of the top 100 DEGs of Infundibulum-Consistent and those of Infundibulum-Inconsistent cells. (H) The results of GO enrichment analysis on the top 1000 DEGs of Infundibulum-Inconsistent cells. The top 10 significant GO terms are illustrated in the figure. (I) The results of KEGG pathway enrichment analysis on the top 1000 DEGs of Infundibulum-Inconsistent cells. The top 20 significant biological pathways are illustrated in the figure. (J) The results of GREAT analysis on the top 1000 DAPs of Infundibulum-Inconsistent cells. The top 20 significantly enriched biological processes are illustrated in the figure. (K) The results of SNPsea analysis on the top 1000 DAPs of Infundibulum-Inconsistent cells. The top 30 significantly enriched tissues are illustrated in the figure.

Furthermore, we extended our comparison to methods that solely use scATAC-seq profiles, including EpiAnno (Chen et al., 2022), SANGO (Zeng et al., 2024), as well as the previously mentioned three conventional machine learning methods. Similarly, MultiKano utilized both omics profiles, whereas the baseline methods only utilized scATAC-seq profiles. As shown in Figs. 2B and S2, MultiKano demonstrated superior performance on all six datasets. Notably, in datasets such as Cortex, Brain, and Kidney, all baseline methods exhibited markedly poor performance. This poor performance is likely attributed to the high noise levels and extreme sparsity inherent in scATAC-seq data, indicating that reliance solely on scATAC-seq profiles does not yield accurate annotation results. In contrast, MultiKano incorporates the relative higher-quality and lower-noise scRNA-seq data, thereby achieving superior annotation performance. Taken together, MultiKano successfully integrates single-cell transcriptomic and chromatin accessibility data to perform cell type annotation on single-cell multi-omics data, outperforming both the approaches tailored to scRNA-seq data and those to scATAC-seq data.

We compared MultiKano with methods utilizing only single-omics profiles in the experiments described above. To further validate the advantages of MultiKano, we expanded the benchmarking to include comparisons with machine learning methods using multi-omics profiles. Specifically, we preprocessed the raw single-cell multi-omics data in the same manner as MultiKano (Text S1) and then concatenated the scRNA-seq profile and scATAC-seq profile from each cell to serve as the input for the machine learning methods. The results indicate that although the concatenating strategy allows these conventional machine learning methods to handle two types of omics profiles simultaneously, their predictive performance still falls short of MultiKano (Figs. 2C and S3). Additionally, we further conducted ablation experiments on the six single-cell multi-omics datasets: (i) comparing the performance of MultiKano using either peak counts or gene activity scores to demonstrate the effectiveness of using peak counts as input for scATAC-seq data (Fig. S4); (ii) comparing the performance of MultiKano with and without data augmentation to demonstrate the effectiveness of data augmentation module in MultiKano (Fig. S5); (iii) comparing the performance of MultiKano with KAN and with MLP to demonstrate the effectiveness of KAN module in MultiKano (Fig. S6). We also provided some intuitive explanations on why KAN performs better than MLP (Text S5). All the results show that each of the modules in MultiKano is a highly effective component that contributes to making MultiKano an accurate annotation method.

Furthermore, considering that datasets from different tissues always contain varying numbers of cell types, we sought to verify whether MultiKano consistently outperformed other methods regardless of the number of cell types in the dataset. To conduct this experiment, we gradually reduced the number of cell types in the dataset by randomly removing one cell type each time, until only two cell types remained. We compared MultiKano against three machine learning methods that utilize multi-omics profiles. Taking the datasets Cortex (Fig. 2D) and SkinA (Fig. S7) as examples, the results demonstrate that as the number of removed cell types increases, the performance of MultiKano remains stable and superior compared to other methods, which highlights the robustness of MultiKano in annotating single-cell multi-omics datasets that encompass a variety of cell types, affirming its effectiveness across diverse biological datasets. In the above experiments, we have systematically demonstrated the superiority of MultiKano via cross-validation, which is widely used and vital for validating model performance. However, in real-world applications, we often train the model on one dataset and make predictions on another, also called inter-dataset annotation. Therefore, we collected a new dataset BMMC (Text S2; Table S1), which consists of multiple batches, to evaluate MultiKano’s performance in inter-dataset annotation. The BMMC dataset was originally generated with nested batch effects by collecting cells from multiple donors across four geographically distinct sites. In other words, the BMMC dataset captures both within-site donor batch variation and site-specific variation. We regarded the cells derived from a single donor at a specific site as one batch, trained the model on one batch, and made predictions on the other batches to better mimic real-world annotation scenarios. Taking the training model on batch SiteA_DonorA as an example, MultiKano demonstrated excellent annotation performance when the test set was sequenced at the same site but a different donor with the training set (Fig. S8A and S8B). The results indicate that MultiKano effectively handles biological batch effects between training and test sets. Furthermore, even when the test set originated from different donors and different sites from the training set, introducing additional variability in both biological and technical aspects, MultiKano still managed to perform accurate cell type annotation (Fig. S8C and S8D). This highlights MultiKano’s robustness and ability to generalize well across datasets with nested batch effects, making it a reliable tool for real-world applications.

In the quantitative experiments mentioned above, we typically rely on the cell type labels provided in the original studies as “Ground truth” for assessing annotation accuracy. However, these “Ground truth” labels are usually derived from manual annotations, which may be subjective and inaccurate. As a result, the so-called “Ground truth” may not accurately represent the true cell types to which the cells belong. Therefore, solely using classification metrics to evaluate the annotation performance might introduce bias. Taking the SkinA dataset as an example, the heatmap plot shows that MultiKano successfully predicts the majority of Infundibulum cells correctly (Fig. S9). In contrast, TOSICA, a recently introduced and high-performing cell type annotation method for scRNA-seq data (Chen et al., 2023), labels nearly 30% of Infundibulum cells as other types (Fig. S9). To facilitate description, we have categorized the cells with “Ground truth” of Infundibulum into two groups: the cells predicted as Infundibulum by both MultiKano and TOSICA are termed as “Infundibulum-Consistent”, while the cells predicted as Infundibulum by MultiKano but as other types by TOSICA are termed as “Infundibulum-Inconsistent”. The existence of Infundibulum-Inconsistent cells could stem from two sources: either the “Ground truth” labels are inaccurate, suggesting that TOSICA’s predictions are correct, or TOSICA itself lacks sufficient precision, indicating that MultiKano’s predictions are correct. To further explore whether Infundibulum-Inconsistent cells truly belong to the Infundibulum type, as predicted by MultiKano, we conducted more in-depth downstream analyses. Firstly, we identified differentially expressed genes (DEGs) in Infundibulum-Consistent cells by comparing their gene expression profiles with those of other cells in the dataset (Text S6). Similarly, we identified DEGs in Infundibulum-Inconsistent cells through the same strategy. We observed that there was a significant overlap between the top 30 DEGs of Infundibulum-Consistent cells and the top 30 DEGs of Infundibulum-Inconsistent cells (Fig. 2E and 2F). To further investigate the overlapping phenomenon, we employed a Venn diagram to visualize the overlap of the top 100 DEGs between the two groups. We observed that 69% of the DEGs overlapped between Infundibulum-Consistent and Infundibulum-Inconsistent cells (Fig. 2G). These findings indicate that even though MultiKano and TOSICA sometimes disagree in their predictions, the gene expression patterns of the cells with inconsistent predictions align more closely with the cell type predicted by MultiKano.

To investigate whether the functional biological characteristics of Infundibulum-Inconsistent cells align with the predictions made by MultiKano, we conducted Gene Ontology (GO) and Kyoto Encyclopedia of Genes and Genomes (KEGG) enrichment analyses on the top 1000 DEGs of the Infundibulum-Inconsistent cells (Text S6). As shown in Fig. 2H, the results of the GO enrichment analysis indicated that the negative regulation of RNA polymerase II transcription plays a significant role in Infundibulum-Inconsistent cells, which is closely related to skin barrier functions and the regulation of the cell cycle. Additionally, the crucial role of signal transduction in regulating cellular responses, proliferation, and differentiation was emphasized, further supporting that the functional characteristics of these cells align with Infundibulum (Kossard, 2021). KEGG pathway analysis revealed the significant roles of cellular senescence and human T-cell leukemia virus 1 infection in these cells (Fig. 2I), which are closely associated with cellular responses to damage, cell cycle regulation, and the onset of skin cancer (El‐Domyati et al., 2010). Overall, these GO and KEGG analysis results suggest that the functional characteristics of the Infundibulum-Inconsistent cells align with the cell type predicted by MultiKano, validating the superior performance of MultiKano and demonstrating its potential in revealing complex biological systems.

We further extended our investigations by employing scATAC-seq profiles to conduct additional downstream analyses (Text S6). Here, we identified the top 1000 differentially accessible peaks (DAPs) in Infundibulum-Inconsistent cells. Leveraging these DAPs, we conducted genomic region enrichment of annotation tool (GREAT) analysis to identify significant pathways associated with the Infundibulum-Inconsistent cells (Text S6; Fig. 2J). We noted that several identified biological processes, such as cell-cell junction organization and adherens junction organization, are vital for maintaining the structural and functional integrity of skin layers. Moreover, processes directly linked to skin formation and epidermal development, like skin development and epidermis development, were also highlighted. These biological processes are highly relevant to the functions of Infundibulum cells (Schneider and Paus, 2014). Additionally, we further conducted single-nucleotide polymorphisms (SNPs) enrichment analysis using SNPsea to obtain tissues explicitly affected by identified DAPs (Text S6). We can see that tissues related to skin are significantly enriched within the DAPs (Fig. 2K). Taken together, the comprehensive results from all the above downstream analyses demonstrate that MultiKano is not only highly effective in annotating cell types but also valuable in unveiling intricate cell-type-specific gene regulatory mechanisms.

In summary, we developed MultiKano, the first automatic cell type annotation method specifically designed for single-cell multi-omics data, which achieves accurate annotation by leveraging the KAN model and a data augmentation technique. Comprehensive experiments on multiple datasets show the advantages of MultiKano compared to not only baseline methods using single-omics profiles but also conventional machine learning methods using multi-omics profiles. Model ablation experiments also show that all the modules in MultiKano can benefit annotation performance. Additionally, we have shown that MultiKano is robust across datasets with varying numbers of cell types, emphasizing its capability to handle complex datasets effectively. More importantly, the inter-dataset annotation experiments demonstrate the promising advantages of MultiKano in practical application scenarios. Finally, through a series of downstream analyses, including GO term enrichment analysis, KEGG pathway enrichment analysis, GREAT analysis and SNP enrichment analysis, MultiKano not only demonstrates its superior performance but also shows its potential in revealing the intricacies of complex biological systems. We also provide several avenues for improving MultiKano, including delving deeper into the integration of multi-omics data and exploring the potential of using unpaired multi-omics datasets to enhance model training.

## Supplementary data

Supplementary data is available at *Protein & Cell* online https://doi.org/10.1093/procel/pwae069.

pwae069_suppl_Supplementary_Material

## Footnotes

S.L., X.H.Z., S.J., Q.Y., and S.C. were responsible for the design, analysis, organization, as well as drafting and revising the manuscript. S.C. and Q.Y. conceived this work. S.C., S.L., X.H.Z., and S.J. implemented and validated MultiKano. S.T. helped design the model. L.Y., H.H., Y.J., X.L.Z. and Y.Z. helped revise the manuscript. S.C. and Q.Y. supervised the entire study. No AI tools had been used in this work.

## Notes

This work was supported by the National Natural Science Foundation of China (Grant Nos. 62203236, 62473212), the Young Elite Scientists Sponsorship Program by CAST (2023QNRC001), the Young Elite Scientists Sponsorship Program by BAST (BYESS2023383), and the Emerging Interdisciplinary Platform for Medicine and Engineering in Sports (EIPMES).

## Notes

The Cortex dataset is available at GEO with the accession number GSE1260744. The Brain, SkinA and SkinB datasets are available at GEO with the accession number GSE140203. The Kidney dataset is available at GEO with the accession number GSE117089. The PBMC dataset is available on the 10x Genomics website. The BMMC dataset is available at GEO with the accession number GSE194122. Source codes and detailed tutorials of MultiKano are freely accessible at GitHub (BioX‐NKU/MultiKano).
