# Supplementary material for: MultiKano: an automatic cell type annotation tool for single-cell multi-omics data based on Kolmogorov–Arnold network and data augmentation
Source: Protein Cell. 2024 Dec 10;16(5):374–80. doi: 10.1093/procel/pwae069 (PMC12120243; doi:10.1093/procel/pwae069)
Supplement: pwae069_suppl_Supplementary_Material [file pwae069_suppl_supplementary_material.pdf]

Supplementary for
MultiKano: an automatic cell type annotation tool for single-cell multi-omics data based on Kolmogorov-Arnold network and data augmentation

Siyu Li[1,†], Xinhao Zhuang[2,†], Songbo Jia[2,†], Songming Tang[1], Liming Yan[3], Heyang Hua[1], Yuhang Jia[1], Xuelin Zhang[2], Yan Zhang[2], Qingzhu Yang[2,*] and Shengquan Chen[1,*]

[1] School of Mathematical Sciences and LPMC, Nankai University, Tianjin 300071, China

[2] Capital University of Physical Education and Sports, Beijing 100191, China

[3] School of Medicine, Tsinghua University, Beijing 100084, China

[*] Corresponding author: chenshengquan@nankai.edu.cn and yangqingzhu@cupes.edu.cn

[†] These authors contributed equally: Siyu Li, Xinhao Zhuang and Songbo Jia

Contents

Supplementary Texts 3

Text S1 3

Text S2 10

Text S3 12

Text S4 14

Text S5 16

Text S6 19

Supplementary Figures 21

Figure S1 21

Figure S2 22

Figure S3 23

Figure S4 24

Figure S5 25

Figure S6 26

Figure S7 27

Figure S8 28

Figure S9 29

Supplementary Table 30

Table S1 30

Supplementary Texts

Text S1: The model of MultiKano

The model of MultiKano is composed of three main modules: the data preprocessing module, the data augmentation module, and the KAN module.

Data preprocessing module

For a given raw paired single-cell multi-omics dataset with scRNA-seq $\mathbf{X}_{\mathrm{r}} \in \mathbb{R}^{n \times g_0}$ and scATAC-seq $\mathbf{X}_{\mathrm{a}} \in \mathbb{R}^{n \times p_0}$, where n denotes the number of cells, g denotes the number of genes in the scRNA-seq data, and p denotes the number of peaks in the scATAC-seq data, we designed preprocessing methods specific to different omics profiles. For the raw cell-by-gene scRNA-seq count matrix $\mathbf{X}_{\mathrm{r}}$, we first normalize the gene expression values for each cell using the following formula:

$$\mathbf{X}_{\mathrm{r}}^{Normalized}[i][j] = \frac{\mathbf{X}_{\mathrm{r}}[i][j]}{\sum_j^g \mathbf{X}_{\mathrm{r}}[i][j]} \times 10^4.$$

Then we perform a logarithmic transformation on the normalized data to mitigate the influence of highly expressed genes, with the following formula:

$$\mathbf{X}_{\mathrm{r}}^{LogTransformed}[i][j] = \log_{10}\left(\mathbf{X}_{\mathrm{r}}^{Normalized}[i][j] + 1\right).$$

Finally, we identify the highly variable genes using the preprocessed data matrix $\mathbf{X}_{\mathrm{r}}^{LogTransformed}$, with the criteria that the retained genes must exhibit a mean expression value between 0.0125 and 3, and a minimum dispersion of 0.5. Following the above preprocessing steps, we obtain the preprocessed matrix $\mathbf{X}_{\mathrm{r}}' \in \mathbb{R}^{n \times g}$.

For the raw cell-by-peak scATAC-seq count matrix $\mathbf{X}_{\mathrm{a}}$, we first binarize the matrix, and filter out low-frequency peaks that appear in a minority of cells. Specifically,

we set a threshold f_{peak} to 0.06, and only retain those peaks that are present in at least $\lceil f_{peak} \times n \rceil$ cells, thus obtaining the data matrix $\mathbf{X}_{\mathrm{a}}^{filtered}$. Then we perform term frequency-inverse document frequency (TF-IDF) transformation (Chen et al., 2022; Cao et al., 2024; Tang et al., 2024) as follows:

$$\mathbf{X}_{\mathrm{a}}^{TF-IDF}[i][j] = \frac{\mathbf{X}_{\mathrm{a}}^{filtered}[i][j]}{\sum_j^p \mathbf{X}_{\mathrm{a}}^{filtered}[i][j]} \log\left(\frac{n}{\sum_i^n \mathbf{X}_{\mathrm{a}}^{filtered}[i][j]}\right),$$

which represents how important the peak j is for cell i. $\mathbf{X}_{\mathrm{a}}^{TF-IDF}$ is then normalized via

$$\mathbf{X}'_{\mathrm{a}}[i][j] = \frac{\mathbf{X}_{\mathrm{a}}^{TF-IDF}[i][j]}{\sqrt{\sum_{j=1}^{p}(\mathbf{X}_{\mathrm{a}}^{TF-IDF}[i][j])^2}}.$$

After the above preprocessing steps, we obtain the preprocessed matrix $\mathbf{X}'_{\mathrm{a}} \in \mathbb{R}^{n \times p}$.

Data augmentation module

To effectively capture cell heterogeneity in the exceedingly noisy single-cell multi-omics data, we further propose a data augmentation module. Specifically, we first split the preprocessed profiles $\mathbf{X}'_{\mathrm{r}}$ and $\mathbf{X}'_{\mathrm{a}}$ by cell type. The profiles for the k-th cell type are denoted as $\mathbf{X}'_{\mathrm{r}\_k}$ and $\mathbf{X}'_{\mathrm{a}\_k}$. For a particular cell c within $\mathbf{X}'_{\mathrm{r}\_k}$ and $\mathbf{X}'_{\mathrm{a}\_k}$, we have the paired profile $\left(\mathbf{x}'_{\mathrm{r}\_k\_c}, \mathbf{x}'_{\mathrm{a}\_k\_c}\right)$. To generate a synthetic cell, we aim to find another cell c', that exhibits biological characteristics similar to cell c. Then we combine the scRNA-seq profile of cell c with the scATAC-seq profile of cell c' to create a pseudo single-cell multi-omics sample, which serves as a synthetic cell for further analysis.

Here, we further describe how to select the cell c' in detail. For a particular cell c belonging to the k-th cell type, we first shuffle all cells of the k-th cell type and obtain the shuffled set. Then we select the cell in the shuffled set with index $c+1$ as the matched cell c'. This means that for the first cell in $\mathbf{X}'_{\mathrm{r}\_k}$, we select the second cell from the shuffled set as its matched cell. Similarly, for the second cell in $\mathbf{X}'_{\mathrm{r}\_k}$, the third cell in the shuffled set is chosen as its matched cell. For the last cell, we select the first cell from the shuffled set as its matched cell. The cell c' also has a corresponding paired profile $\left(\mathbf{x}'_{\mathrm{r}\_k\_c'}, \mathbf{x}'_{\mathrm{a}\_k\_c'}\right)$. Using cells c and c' from the same cell type, we create a synthetic cell $\left(\hat{\mathbf{x}}'_{\mathrm{r}\_k\_c}, \hat{\mathbf{x}}'_{\mathrm{a}\_k\_c}\right) = \left(\mathbf{x}'_{\mathrm{r}\_k\_c}, \mathbf{x}'_{\mathrm{a}\_k\_c'}\right)$, and denote it as the synthetic cell corresponding to the c-th cell. The core principle of this pairing strategy is that two cells from the same type share comparable biological traits, allowing their distinct omics profiles to match with each other. Based on the pairing strategy, we create synthetic cells for all real cells of each cell type, and obtain the synthetic training set $\widehat{\mathbf{X}}'_{\mathrm{r}}$ and $\widehat{\mathbf{X}}'_{\mathrm{a}}$. Finally, we combine the real cells and synthetic cells to obtain the final training set $\widetilde{\mathbf{X}}'_{\mathrm{r}} \in \mathbb{R}^{2n\times g}$ and $\widetilde{\mathbf{X}}'_{\mathrm{a}} \in \mathbb{R}^{2n\times p}$, effectively increasing the size of the training set and helping the model better distinguish different cell types.

The KAN module

In the KAN module, MultiKano leverages a Kolmogorov–Arnold Network (KAN) (Liu et al., 2024) to perform automatic cell type annotation. By employing learnable activation functions along the network's edges, KAN achieves excellent flexibility and generalizability, enabling efficient learning of complex nonlinear mappings and reducing the risk of overfitting.

KAN is inspired by the Kolmogorov-Arnold representation theorem (Liu et al., 2024). To facilitate a better understanding of KAN, we first bring a brief introduction to the Kolmogorov-Arnold representation theorem. Vladimir Arnold and Andrey Kolmogorov established that if f is a multivariate continuous function on a bounded domain, then f can be written as a finite composition of continuous functions of a single variable and the binary operation of addition. More specifically, for a smooth $f: [0,1]^n \to \mathbb{R}$,

$$f(\mathbf{x}) = f(x_1, \cdots x_n) = \sum_{q=1}^{2n+1} \Phi_q \left(\sum_{p=1}^{n} \phi_{q,p}(x_p) \right),$$

where $\phi_{q,p}: [0,1] \to \mathbb{R}$ and $\Phi_q: \mathbb{R} \to \mathbb{R}$. The formula shows that every multivariate function can be written as the summation of univariate functions. Suppose we have a supervised learning task consisting of input-output pairs $\{\mathbf{x}_i, y_i\}$, where we want to find f such that $y_i \approx f(\mathbf{x}_i)$ for all data points. The Kolmogorov-Arnold representation theorem implies that we are done if we can find appropriate univariate functions $\phi_{q,p}$ and Φ_q. This inspires us to design a neural network which explicitly parametrizes the theorem. Since all functions to be learned are univariate functions, we can parametrize each 1D function as a B-spline basis function. B-spline basis functions are typically constructed recursively using the Cox-de Boor recursion formula:

$$N_{i,0}(t) = \begin{cases} 1, \text{if } t_i \le t < t_{i+1} \\ 0, \text{otherwise} \end{cases},$$

$$N_{i,k}(t) = \frac{t - t_i}{t_{i+k} - t_i} N_{i,k-1}(t) + \frac{t_{i+k+1} - t}{t_{i+k+1} - t_{i+1}} N_{i+1,k-1}(t),$$

where t is the parameter value, t_i are the knots, and k is the order of spline.

Based on the Kolmogorov-Arnold theorem, we further provide the mathematical formulation of the KAN. We represent the shape of KAN by an integer array

$[n_0, n_1, \cdots, n_L]$, where n_l is the number of nodes in the l-th layer in the network. We denote the i-th neuron in the l-th layer by (l, i), and the activation value of the (l, i) neuron value by $x_{l,i}$. Between layer l and layer $l+1$, there are $n_l \times n_{l+1}$ activation functions: the activation function that connects (l, i) and $(l+1, j)$ is denoted by $\phi_{l,j,i}$, where $l = 0, \cdots, L-1, i = 1, \cdots, n_l, j = 1, \cdots, n_{l+1}$. The pre-activation of $\phi_{l,j,i}$ is simply $x_{l,i}$; the post-activation of $\phi_{l,j,i}$ is denoted by $\tilde{x}_{l,j,i} \equiv \phi_{l,j,i}(x_{l,i})$. The activation value of the $(l+1, j)$ neuron is simply the sum of all incoming post-activations:

$$x_{l+1,j} = \sum_{i=1}^{n_l} \tilde{x}_{l,j,i} = \sum_{i=1}^{n_l} \phi_{l,j,i}(x_{l,i}), j = 1, \cdots, n_{l+1}.$$

In matrix form, this reads

$$\mathbf{x}_{l+1} = \underbrace{\begin{pmatrix} \phi_{l,1,1}(\cdot) & \cdots & \phi_{l,1,n_l}(\cdot) \\ \vdots & \ddots & \vdots \\ \phi_{l,n_{l+1},1}(\cdot) & \cdots & \phi_{l,n_{l+1},n_l}(\cdot) \end{pmatrix}}_{\mathbf{\Phi}_l} \mathbf{x}_l,$$

where $\mathbf{\Phi}_l$ is the function matrix corresponding to the l-th KAN layer. A general KAN network is a composition of L layers: given an input vector $\mathbf{x}_0$, the output of KAN is

$$\text{KAN}(\mathbf{x}_0) = (\mathbf{\Phi}_{L-1} \circ \mathbf{\Phi}_{L-2} \circ \cdots \circ \mathbf{\Phi}_1 \circ \mathbf{\Phi}_0)\mathbf{x}_0.$$

Specifically, the KAN model in MultiKano contains an input layer, two hidden layers, and an output layer. During the training process of KAN, we concatenate the scRNA-seq and scATAC-seq profiles of each cell in the final training set $\tilde{\mathbf{X}}'_{\text{r}}$ and $\tilde{\mathbf{X}}'_{\text{a}}$ to serve as the input of KAN. That is, for a cell with its scRNA-seq profile denoted as $\tilde{\mathbf{x}}'_{\text{r}} = (\tilde{x}'_{\text{r},1}, \cdots, \tilde{x}'_{\text{r},g})$, where g is the number of genes, and its scATAC-seq profile denoted as $\tilde{\mathbf{x}}'_{\text{a}} = (\tilde{x}'_{\text{a},1}, \cdots, \tilde{x}'_{\text{a},p})$, where p is the number of peaks, we concatenate the two profiles as:

$$\tilde{\mathbf{x}}' = \left(\tilde{x}'_{\mathrm{r},1}, \cdots, \tilde{x}'_{\mathrm{r},g}, \tilde{x}'_{\mathrm{a},1}, \cdots, \tilde{x}'_{\mathrm{a},p}\right).$$

Then we input the concatenated vector to KAN and obtain the prediction probability of the cell belonging to each cell type from the output layer.

The number of nodes in the input layer is equal to the total number of features of $\tilde{\mathbf{X}}'_{\mathrm{r}}$ and $\tilde{\mathbf{X}}'_{\mathrm{a}}$, while the number of nodes in the output layer is equal to the number of cell types in the training set. The hidden layers consist of 256 and 128 nodes, respectively. Batch normalization is applied after the first layer to stabilize training, while a dropout rate of 0.5 is used after the second layer to mitigate overfitting. This configuration supports effective pattern recognition from multi-omics data while ensuring robustness and generalizability. The network is trained using cross-entropy as the loss function, with the Adam optimizer and a weight decay of 1E-5. Furthermore, a cosine annealing learning rate scheduler is employed to gradually adjust the learning rate, facilitating improved convergence during training.

Additionally, given that the final training set comprises both real and synthetic cells, we have incorporated a loss weight in the loss function to prevent the synthetic cells from diverging excessively from the real cells, thereby ensuring more effective model training:

$$Loss = -\sum_{c=1}^{N} \lambda_c \left(\sum_{k=1}^{K} p_{ck} \log(p_{ck}) \right),$$

where N is the number of cells in the final training set which includes real and synthetic cells, K is the number of cell types, and p_{ck} represents the prediction probability of the c-th cell belonging to the k-th cell type. We add a loss weight λ_c to tailor the loss function based on whether the cell is real or synthetic. For each cell c,

if the cell is a real cell, we set λ_c to 1, reflecting a baseline influence on the model's learning process. Conversely, if the c-th cell is a synthetic cell, we set λ_c as the cosine similarity between the synthetic cell c and its corresponding real cell:

$$\lambda_c = \begin{cases} 1, & \text{if the } c - \text{th cell is a real cell} \\ \cos\left(\mathbf{x}_c^{\text{synthetic}}, \mathbf{x}_c^{\text{real}}\right), & \text{otherwise} \end{cases}'$$

where $\mathbf{x}_c^{\text{synthetic}}$ is the concatenation of the scRNA-seq and scATAC-seq profiles of the synthetic cell c, $\mathbf{x}_c^{\text{real}}$ is the concatenation of the scRNA-seq and scATAC-seq profiles of the corresponding real cell. The more similar the synthetic cell is to the real cell, the greater its contribution to the loss function.

Text S2: Data collection and accessibility

We collected six paired single-cell multi-omics datasets from different species, tissues, and protocols to comprehensively evaluate the performance of MultiKano. The Cortex dataset is derived from the adult mouse cerebral cortices by SNARE-seq, which is available at GEO with the accession number GSE1260744 [https://www.ncbi.nlm.nih.gov/geo/query/acc.cgi?acc=GSE126074] (Chen et al., 2019). The Brain dataset is derived from adult mouse brain by SHARE-seq (Ma et al., 2020). The SkinA and SkinB datasets are both derived from adult mouse skin by SHARE-seq (Ma et al., 2020). All the three datasets profiled by SHARE-seq are available at GEO with the accession number GSE140203 [https://www.ncbi.nlm.nih.gov/geo/query/acc.cgi?acc=GSE140203]. The Kidney dataset is sourced from adult mouse whole kidney by sci-CAR, which is available at GEO with the accession number GSE117089 [https://www.ncbi.nlm.nih.gov/geo/query/acc.cgi?acc=GSE117089] (Cao et al., 2018). The PBMC dataset is composed of human peripheral blood mononuclear cells profiled by 10x-Multiome, which can be accessed at https://support.10xgenomics.com/single-cell-gene-expression/datasets/3.0.0/pbmc_10k_v3 (Luecken et al., 2021). Furthermore, to perform the inter-dataset annotation, we collected another BMMC dataset, which is derived from the human bone marrow profiled by 10x-Multiome (Luecken et al., 2021). The BMMC dataset was originally generated with nested batch effects by collecting cells from multiple donors across four geographically distinct sites. In other words, the BMMC dataset captures both within-site donor batch variation, as sequencing was

performed on multiple unique donors at each site, and site-specific variation, as sequencing was performed across different sites for each donor. The BMMC dataset is available at GEO with the accession number GSE194122 [https://www.ncbi.nlm.nih.gov/geo/query/acc.cgi?acc=GSE194122].

Text S3: Details of evaluation metrics

For the evaluation of cell type annotation performance, we assessed the results with three metrics: Accuracy, Cohen's kappa value (Kappa), and macro F1 score (F1-macro), all of which have been suggested in the recent benchmark studies (Abdelaal et al., 2019; Ma et al., 2021). Assuming that there are N cells with ground-truth cell types denoted as $\mathbf{T} = \{T_1, \dots\dots, T_N\}$ and annotated cell types denoted as $\mathbf{P} = \{P_1, \dots\dots, P_N\}$, Accuracy can be calculated as follows:

$$\text{Accuracy} = \frac{\sum_{i=1}^{N} I(T_\text{i} = P_\text{i})}{N},$$

where $I(\cdot)$ represents the indicator function. The indicator function returns 1 if the annotated cell type matches the ground-truth cell type for cell i (i.e., $T_\text{i} = P_\text{i}$), and it returns 0 otherwise.

Kappa further takes the possibility of the agreement occurring by chance into account, which can be calculated as follows:

$$\text{Kappa} = \frac{\text{Accuracy} - p_e}{1 - p_e},$$

where p_e is the hypothetical probability of chance variable calculated as follows:

$$p_e = \frac{1}{N^2} \sum_{k=1}^{K} a_k b_k,$$

where a_k and b_k are the number of the k-th cell type in $\mathbf{T}$ and $\mathbf{P}$, respectively. K is the total number of cell types.

F1-macro are derived from F1-score:

$$\text{F1}(k) = 2 \times \frac{precision_k \times recall_k}{precision_k + recall_k},$$

where $precision_k$ is the number of true positive samples divided by the number of all samples identified as the k-th cell type, while $recall_k$ is the number of true positive samples divided by the number of samples in the k-th cell type. Then we have the following F1-macro:

$$\mathrm{F1-macro} = \frac{1}{K}\sum_{k=1}^{K} \mathrm{F1}(k),$$

where $\mathrm{F1}(k)$ represents the F1-score of the k-th cell type, and K is the number of cell types.

Text S4: Baseline methods

We compared the performance of MultiKano with seven baseline methods, including two methods specially designed for scRNA-seq data, two methods for scATAC-seq data, and three machine learning methods. The two methods designed for scRNA-seq data are scPred (Alquicira-Hernandez et al., 2019) and TOSICA (Chen et al., 2023). scPred is an automatic cell type annotation method to classify single cells based on singular value decomposition and a support vector machine model, and has demonstrated superior performance in several studies (Abdelaal et al., 2019; Ma et al., 2021; Chen et al., 2023). We input the raw scRNA-seq training set to train scPred and obtained the annotation results on the test set. All steps followed the tutorials provided on the GitHub [https://github.com/powellgenomicslab/scPred]. TOSICA is a recently proposed high-performing method, which employs a multi-head self-attention network for interpretable cell type annotation in scRNA-seq data (Chen et al., 2023). Similarly, we input the raw scRNA-seq training set to train TOSICA and obtained the annotation results on the test set, following the provided tutorials on the GitHub [https://github.com/JackieHanLab/TOSICA]. All other parameters were set to the default values. For scATAC-seq data, the methods assessed are EpiAnno (Chen et al., 2022) and SANGO (Zeng et al., 2024). EpiAnno is the first automatic cell type annotation method tailored to scATAC-seq data, which utilizes a Bayesian neural network to perform annotation (Chen et al., 2022). We input the raw cell-by-peak scATAC-seq data to EpiAnno and obtained the annotation results. SANGO is an accurate and scalable graph-based method for annotating cells within scATAC-seq data

by integrating DNA sequence information (Zeng et al., 2024). We input the raw cell-by-peak scATAC-seq data and the corresponding DNA sequence to SANGO and obtained the annotation results. We implemented the two methods following their tutorials on the GitHub [https://github.com/xy-chen16/EpiAnno, https://github.com/cquzys/SANGO]. The three machine learning methods evaluated are Support Vector Machine (SVM) with a linear kernel, Random Forest (RF), and Multi-Layer Perceptron (MLP), all recommended by two benchmark studies for identifying cell types (Abdelaal et al., 2019; Ma et al., 2021). To build the machine learning models, we used the scikit-learn (version: 1.1.2) package. We used sklearn.svm.LinearSVC() for SVM, sklearn.ensemble.RandomForestClassifier() for RF, and sklearn.neural_network.MLPClassifier() for MLP. All the above supervised methods do not require additional information except for annotated cell type labels in training sets.

Text S5: Intuitive explanations on why KAN performs better than MLP

Kolmogorov-Arnold Network (KAN) is designed based on the Kolmogorov-Arnold representation theorem, which demonstrates that any continuous multi-variable function can be represented as a composition of functions of only one variable. This foundational principle empowers KAN to effectively capture and represent complex, high-dimensional data mappings. Here we further provide some more intuitive explanations on why KAN performs better than MLP.

Firstly, KAN possesses superior function approximation capabilities compared to MLP. KAN is built upon the Kolmogorov-Arnold representation theorem, which asserts that any continuous multi-variable function can be decomposed into a finite combination of univariate functions. This enables KAN to flexibly and robustly model complex non-linear interactions within data, enhancing its function approximation capabilities. In contrast, while MLP can theoretically approximate any continuous function, it requires substantially deeper or broader network architectures. Therefore, when dealing with complex functional mappings, KAN offers better approximation accuracy and flexibility than traditional MLP. This advantage becomes particularly pronounced when handling with extremely high-dimensional single-cell datasets.

Secondly, compared to MLP, KAN can avoid issues such as gradient vanishing and the entrapment in local optima. One of the unique features of KAN is its ability to decompose complex, high-dimensional inputs into simpler, single-variable functions. This technique facilitates the independent learning of local features, which are subsequently integrated to effectively discern global patterns. The structured

decomposition and combination approach allows KAN to more efficiently comprehend and model the overarching objectives of the data, resulting in a more stable gradient and a more efficient optimization process. However, MLP attempts a direct global mapping of high-dimensional inputs, which can lead to challenges such as gradient vanishing and the entrapment in local optima.

Thirdly, KAN reduces model complexity compared to MLP. Leveraging its function decomposition-based structure, KAN requires fewer layers and parameters to effectively represent complex data. This reduction in model complexity not only diminishes the computational burden but also lowers the demand for computational resources during training, thus boosting overall efficiency. In contrast, MLP often necessitates a multitude of hidden layers and neurons to capture complex data patterns, potentially causing inefficiencies, particularly throughout training phases.

Furthermore, many studies across various tasks have successfully replaced MLP with KAN and consistently achieved superior performance. For example, Nehma et al. conducted a comparison of the performance of KAN and MLP in the context of learning Koopman operators with control. KAN was found to be superior in nearly all aspects of training; learning 31 times faster, being 15 times more parameter efficient and predicting 1.25 times more accurately as compared to MLP (Nehma and Tiwari, 2024). Cheon demonstrated the effectiveness of KAN for vision tasks through multiple trials on the MNIST, CIFAR10, and CIFAR100 datasets, and suggest that KAN holds significant promise for vision tasks (Cheon, 2024). Liu et al. explored the incorporation of KAN into the actor and critic networks for offline reinforcement learning (RL) and

demonstrated that KAN can achieve performance close to the commonly used MLP with significantly fewer parameters (Guo et al., 2024). In summary, KAN serves as a strong alternative to MLP for many tasks, but it has not been utilized in the context of single-cell data modeling previously. In this study, we applied KAN to single-cell data modeling for the first time and achieved promising results.

Text S6: Implementation details of downstream analyses

We identified the differentially expressed genes (DEGs) and differentially accessible peaks (DAPs) using the Scanpy (Wolf et al., 2018) pipeline.

Enrichment analysis of DEGs

We used the DEGs to perform gene enrichment analysis using the DAVID tool (Huang et al., 2009; Sherman et al., 2022), covering both Gene Ontology (GO) (Consortium, 2019) and Kyoto Encyclopedia of Genes and Genomes (KEGG) (Kanehisa and Goto, 2000) enrichment analyses. The GO terms include molecular function (MF), biological process (BP), and cellular component (CC). The top 10 significantly enriched GO terms and the top 20 significantly enriched KEGG pathways are illustrated.

GREAT analysis

Genomic Regions Enrichment of Annotations Tool (GREAT) analysis is a bioinformatics approach used to link genomic regions to known biological pathways and functions (McLean et al., 2010). We submitted the DAPs to the GREAT server with the default settings to identify significant biological processes associated with the DAPs and thus obtain functional insights for the corresponding cell subpopulation. The top 20 significant biological processes are illustrated.

SNPsea analysis

SNPsea is an enrichment algorithm designed for analyzing single-nucleotide polymorphisms (SNPs) to pinpoint specific cell types, tissues, and biological pathways

that are influenced by risk loci associated with traits (Slowikowski et al., 2014). It tests trait-associated genomic loci for enrichment of specificity to conditions (cell types, tissues and pathways). We quantified the enrichments of DAPs in tissue-specific accessibility profiles across 79 tissues. The top 30 significantly enriched tissues are illustrated.

Supplementary Figures

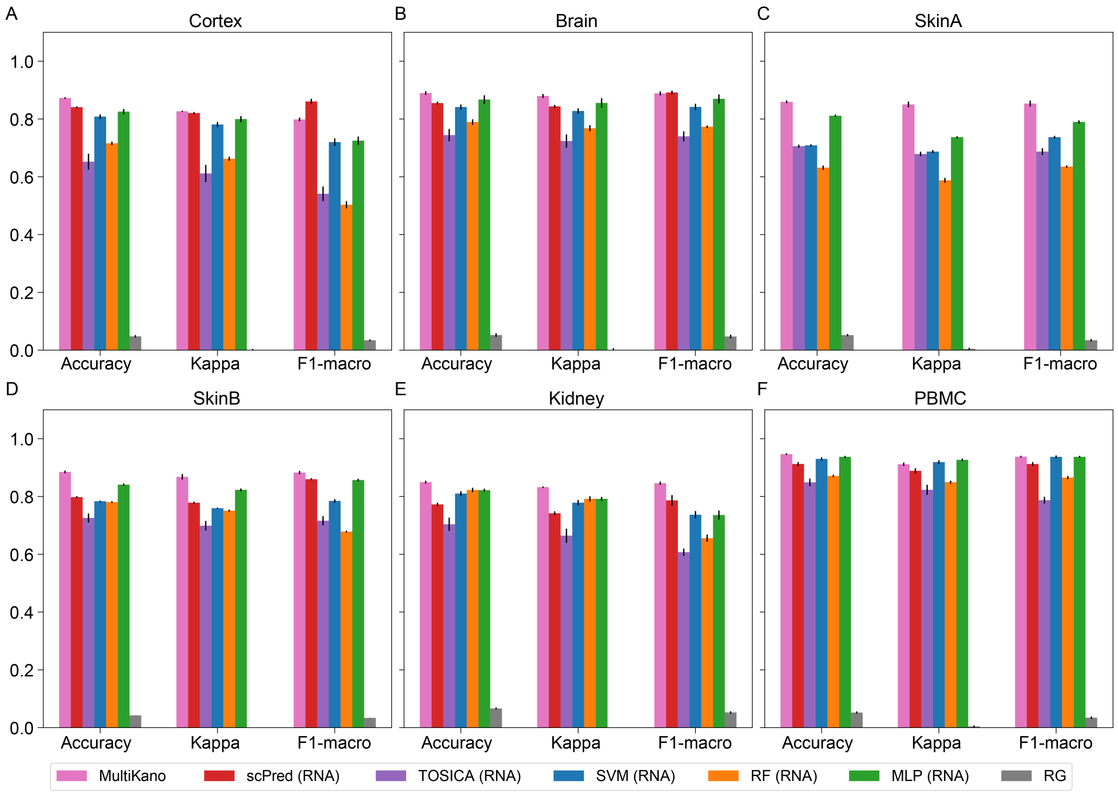

Figure S1. Annotation performance of MultiKano and baseline methods for scRNA-seq data as well as Random Guessing (RG) across six single-cell multi-omics datasets. scPred encountered an error "line search fails" on the SkinA dataset.

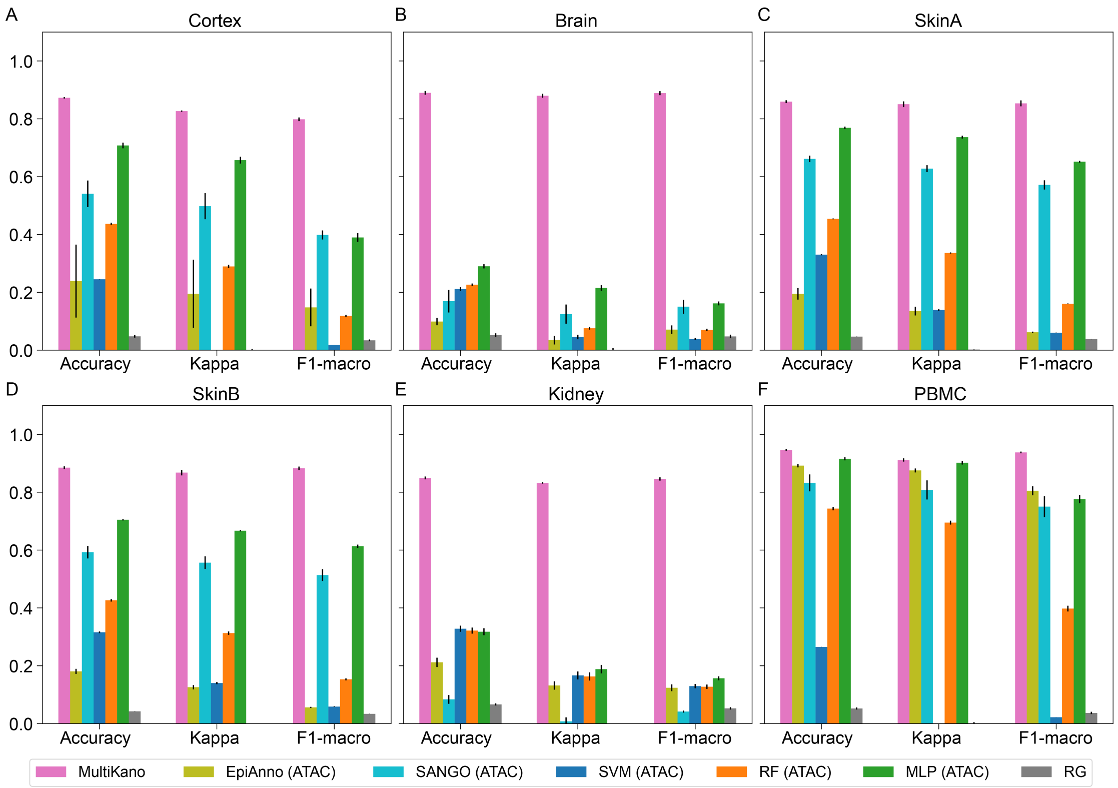

Figure S2. Annotation performance of MultiKano and baseline methods for scATAC-seq data as well as Random Guessing (RG) across six single-cell multi-omics datasets.

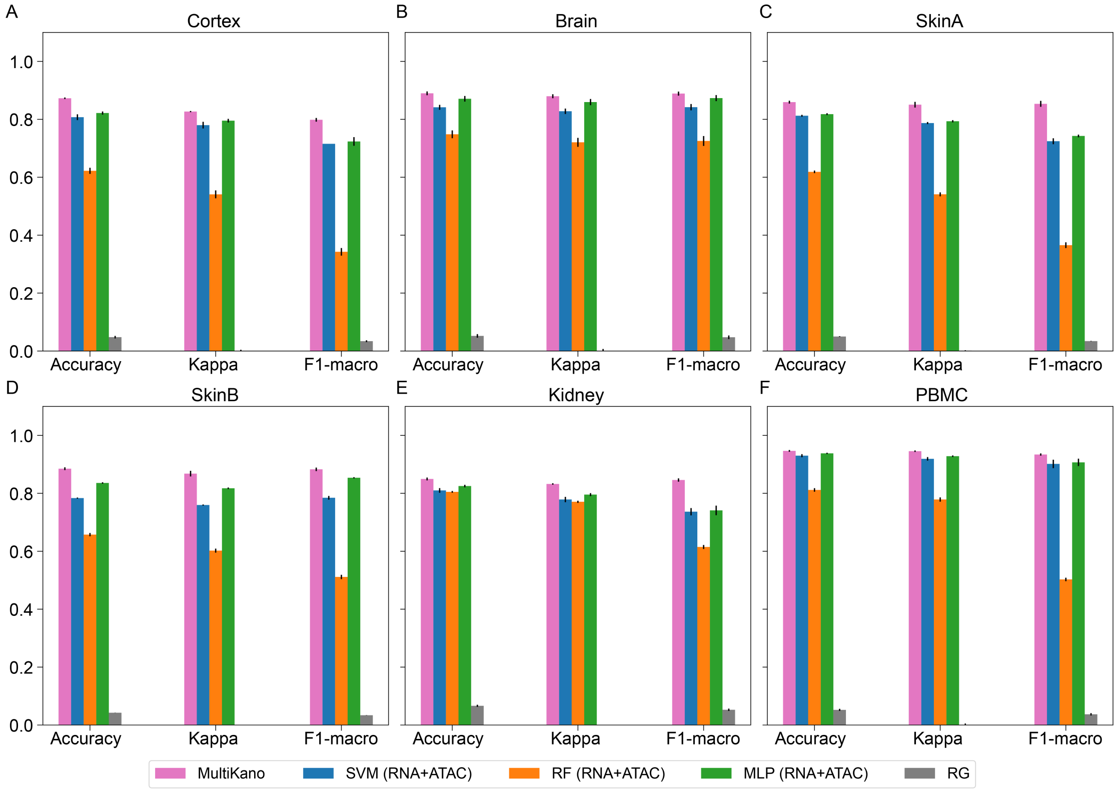

Figure S3. Annotation performance of MultiKano and conventional machine learning methods using multi-omics data as well as Random Guessing (RG) across six single-cell multi-omics datasets.

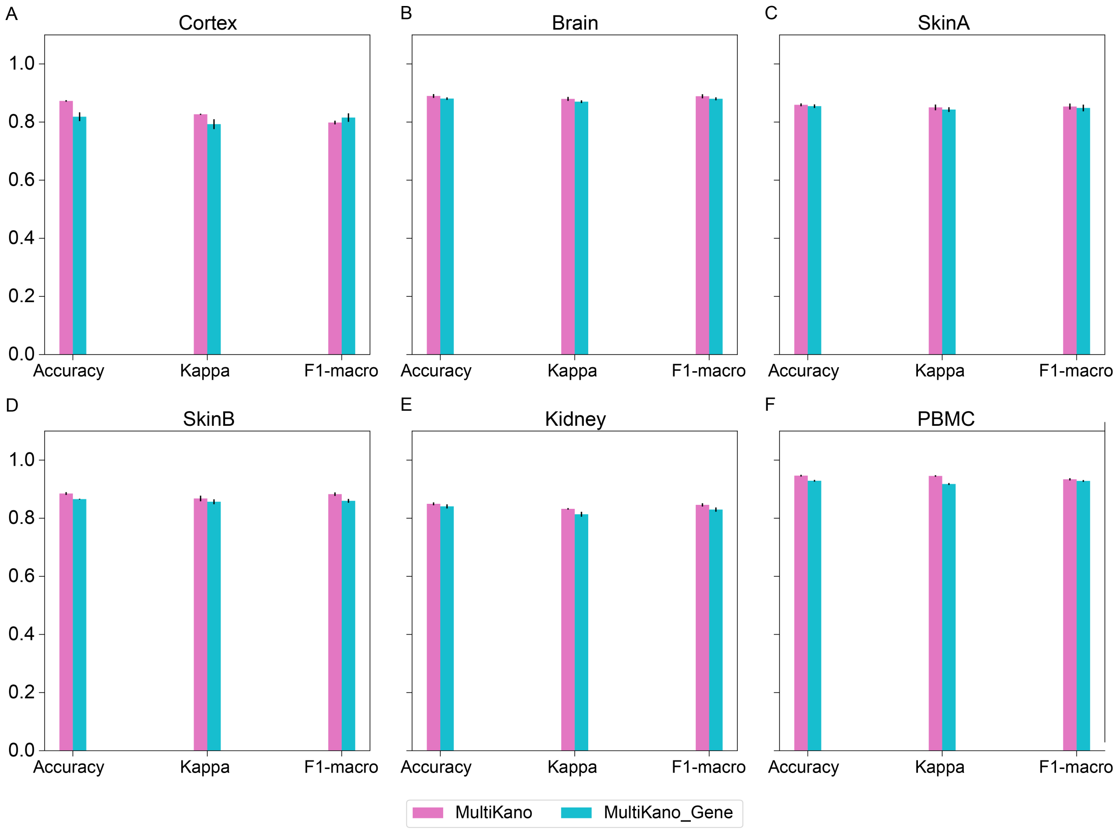

Figure S4. Annotation performance of MultiKano and MultiKano_Gene across six single-cell multi-omics datasets. MultiKano_Gene represents that utilizing gene activity scores as input for scATAC-seq data.

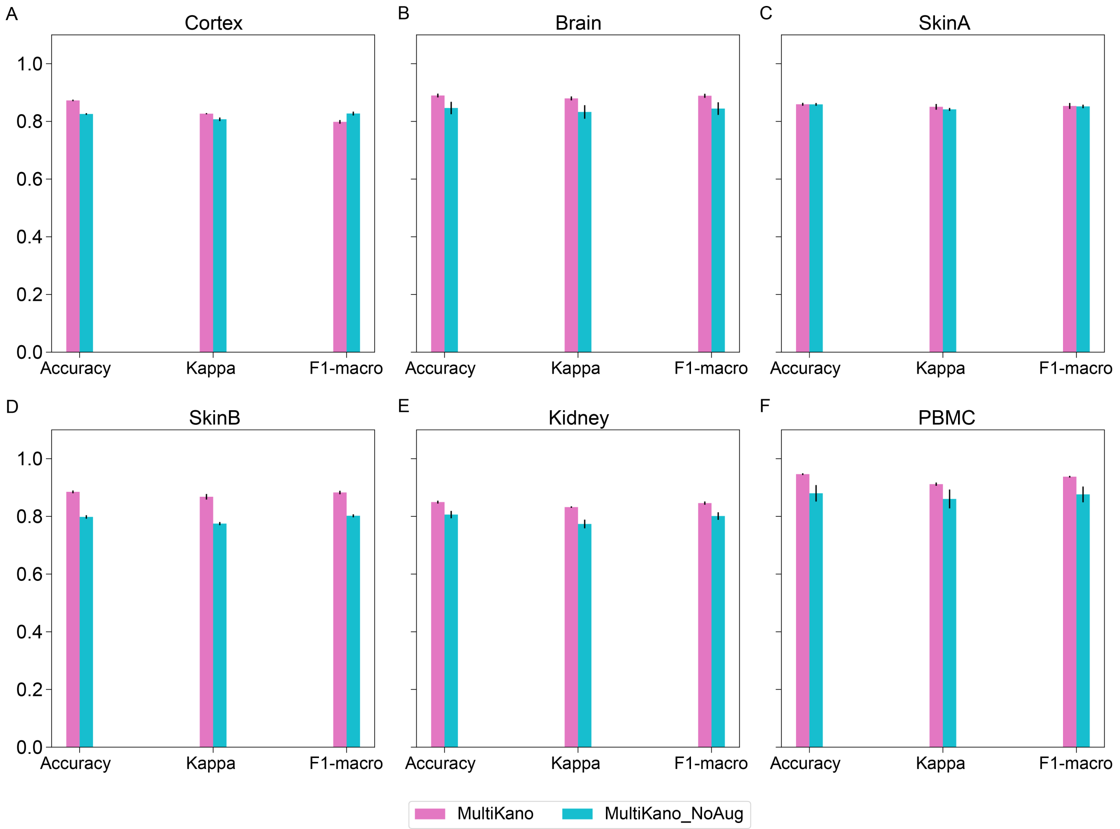

Figure S5. Annotation performance of MultiKano and MultiKano_NoAug across six single-cell multi-omics datasets. MultiKano_NoAug represents MultiKano without data augmentation module.

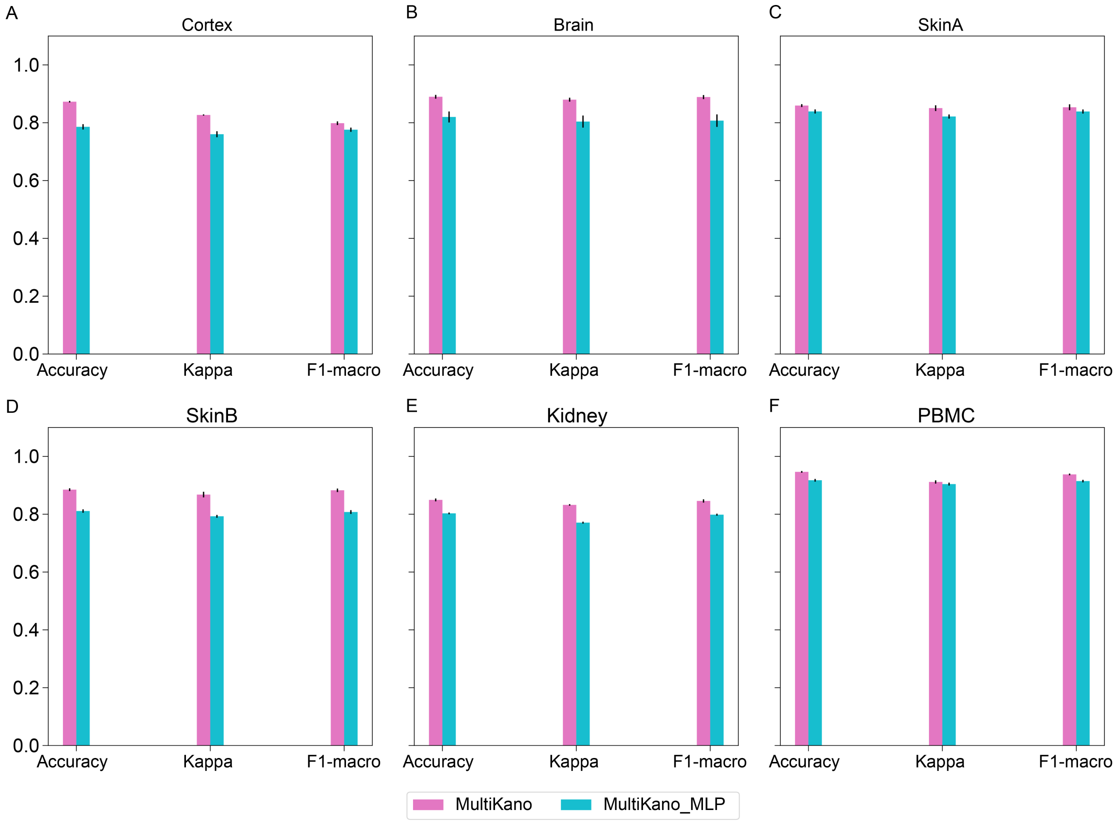

Figure S6. Annotation performance of MultiKano and MultiKano_MLP across six single-cell multi-omics datasets. MultiKano_MLP represents replacing KAN in MultiKano with MLP.

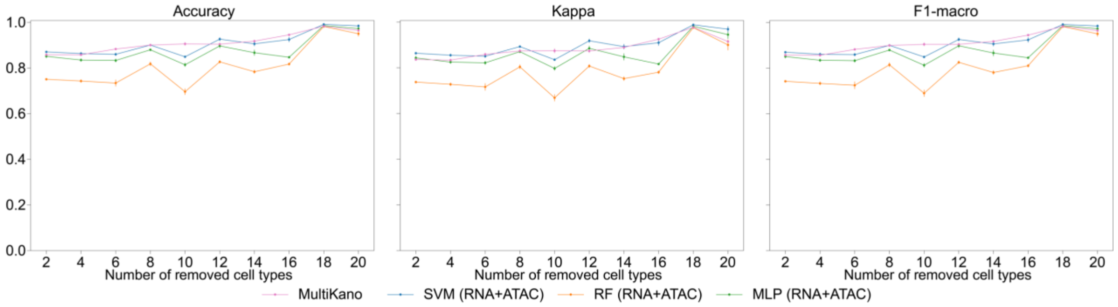

Figure S7. The robustness of MultiKano and conventional machine learning methods using multi-omics data to the number of cell types on the SkinA dataset.

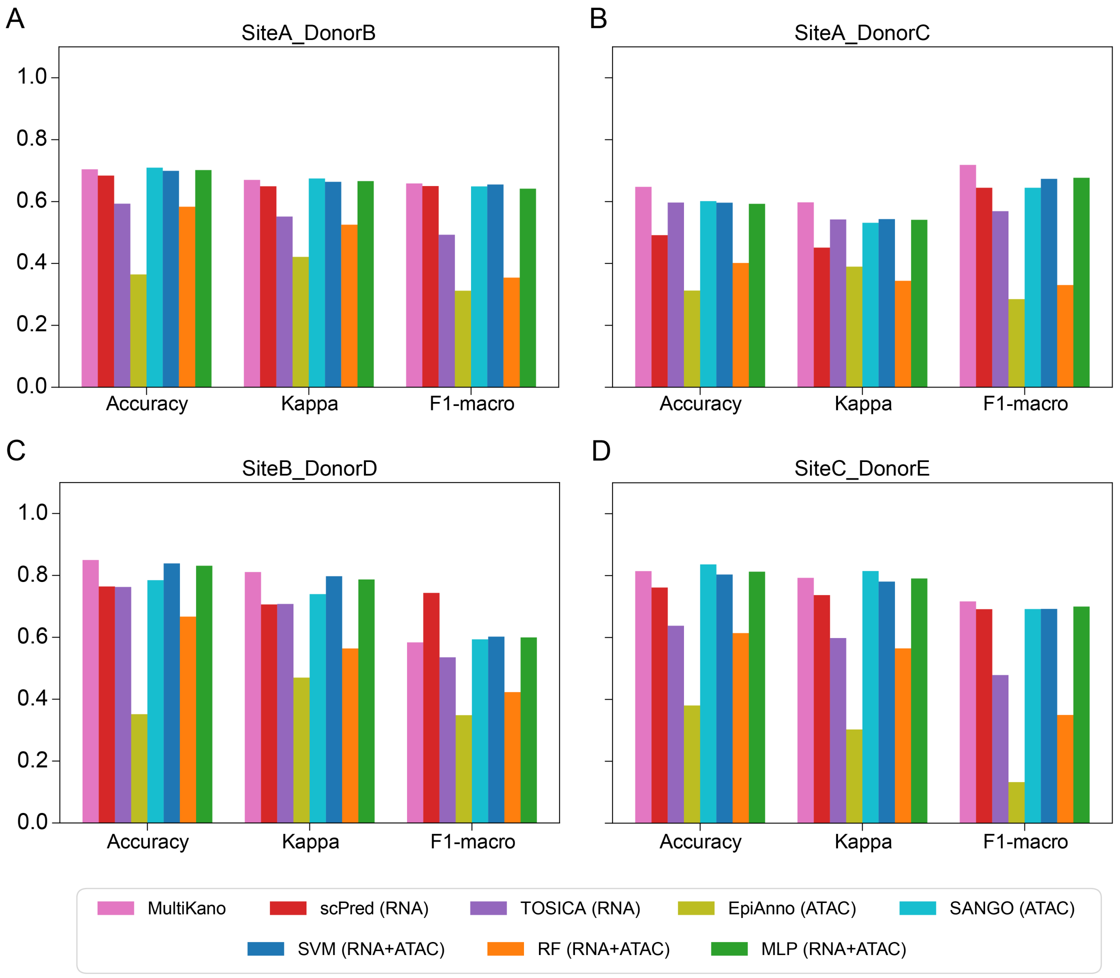

Figure S8. Annotation performance of MultiKano in inter-dataset annotation. The model is trained on the batch SiteA_DonorA. The title of each subgraph represents the test batch.

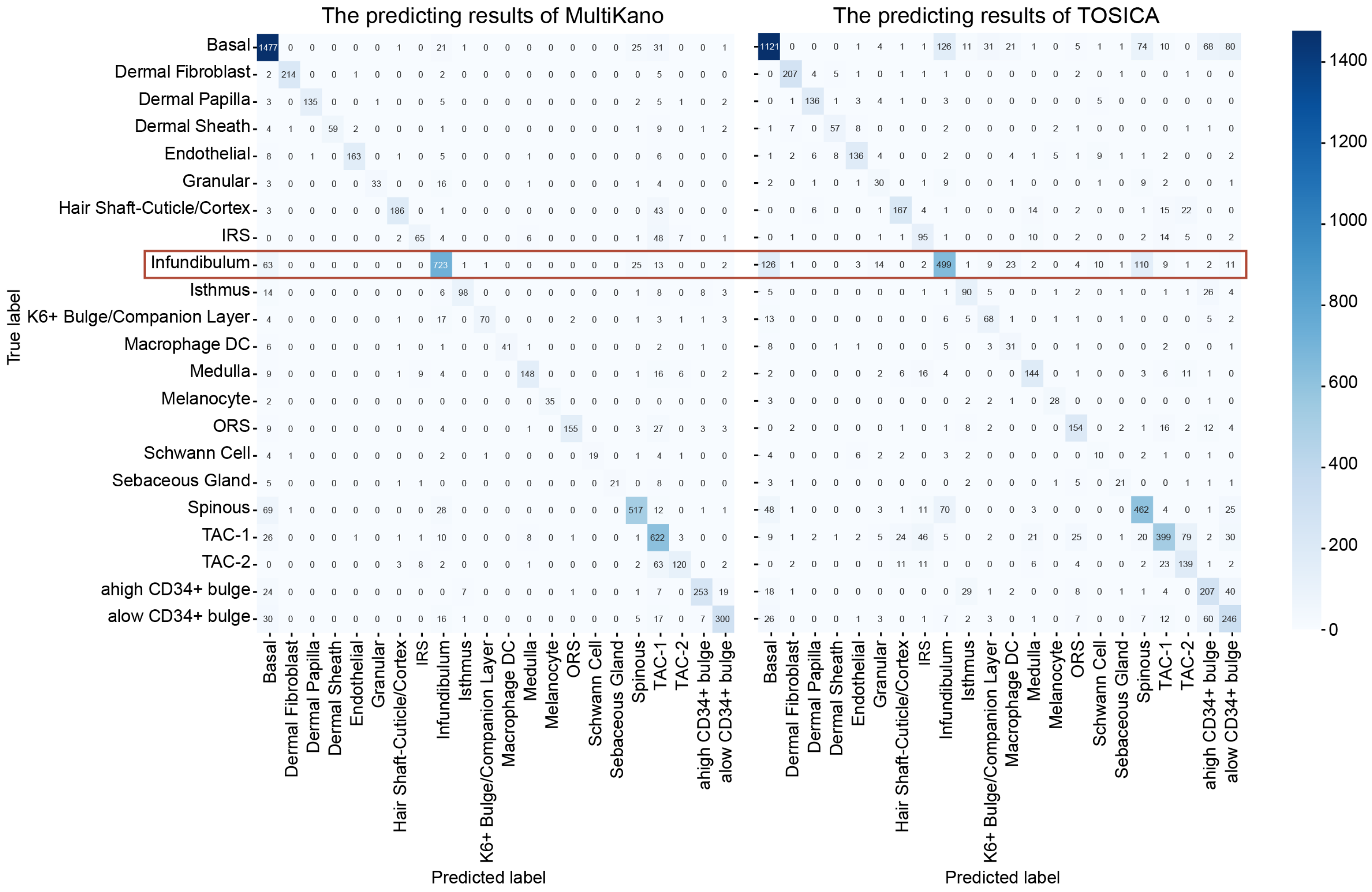

Figure S9. Cell type annotation results of MultiKano and TOSICA on the SkinA dataset.

Supplementary Table

Table S1. A summary of the datasets for evaluating the performance of MultiKano, including the detailed information about the number of cells, features, and cell types, as well as the imbalance degree of cell types, sparsity, species, and protocol.

Data name	No. of cells	No. of genes	No. of peaks	No. of cell types	Proportion of major type	Imbalance degree	RNA sparsity	ATAC sparsity	Species	Protocol
Cortex	9190	28930	241757	22	0.245	0.194	0.968	0.990	*Mus musculus*	SNARE-seq
Brain	3293	21127	428041	19	0.179	0.065	0.922	0.998	*Mus musculus*	SHARE-seq
SkinA	32231	21478	340341	22	0.242	0.164	0.971	0.988	*Mus musculus*	SHARE-seq
SkinB	34774	23296	344592	23	0.224	0.152	0.972	0.988	*Mus musculus*	SHARE-seq
Kidney	8837	49584	252741	14	0.226	0.163	0.987	0.997	*Mus musculus*	sci-CAR
PBMC	9631	29095	107194	19	0.265	0.198	0.934	0.931	*Homo sapiens*	10x-Multiome
BMMC	69249	13431	116490	22	0.167	0.760	0.917	0.969	*Homo sapiens*	10x-Multiome

Supplementary References

Abdelaal T, Michielsen L, Cats D, et al. A comparison of automatic cell identification methods for single-cell RNA sequencing data. Genome Biol 2019; **20**(1): 1-19.

Alquicira-Hernandez J, Sathe A, Ji HP, et al. scPred: Accurate supervised method for cell-type classification from single-cell RNA-seq data. Genome Biol 2019; **20**(1): 1-17.

Cao J, Cusanovich DA, Ramani V, et al. Joint profiling of chromatin accessibility and gene expression in thousands of single cells. Science 2018; **361**(6409): 1380-1385.

Cao Y, Zhao X, Tang S, et al. scButterfly: a versatile single-cell cross-modality translation method via dual-aligned variational autoencoders. Nat Commun 2024; **15**(1): 2973.

Chen J, Xu H, Tao W, et al. Transformer for one stop interpretable cell type annotation. Nat Commun 2023; **14**(1): 223.

Chen S, Lake BB and Zhang K. High-throughput sequencing of the transcriptome and chromatin accessibility in the same cell. Nat Biotechnol 2019; **37**(12): 1452-1457.

Chen X, Chen S, Song S, et al. Cell type annotation of single-cell chromatin accessibility data via supervised Bayesian embedding. Nat Mach Intell 2022; **4**(2): 116-126.

Cheon M. Demonstrating the efficacy of kolmogorov-arnold networks in vision tasks. arXiv preprint arXiv:2406.14916 2024.

Consortium GO. The gene ontology resource: 20 years and still GOing strong. Nucleic Acids Res 2019; **47**(D1): D330-D338.

Guo H, Li F, Li J, et al. KAN vs MLP for offline reinforcement learning. arXiv preprint arXiv:2409.09653 2024.

Huang DW, Sherman BT and Lempicki RA. Systematic and integrative analysis of large gene lists using DAVID bioinformatics resources. Nat Protoc 2009; **4**(1): 44-57.

Kanehisa M and Goto S. KEGG: kyoto encyclopedia of genes and genomes. Nucleic Acids Res 2000; **28**(1): 27-30.

Liu Z, Wang Y, Vaidya S, et al. Kan: Kolmogorov-arnold networks. arXiv preprint arXiv:2404.19756 2024.

Luecken MD, Burkhardt DB, Cannoodt R, et al. A sandbox for prediction and integration of DNA, RNA, and proteins in single cells. Thirty-fifth conference on neural information processing systems datasets and benchmarks track (Round 2) 2021.

Ma S, Zhang B, LaFave LM, et al. Chromatin potential identified by shared single-cell profiling of RNA and chromatin. Cell 2020; **183**(4): 1103-1116.e1120.

Ma W, Su K and Wu H. Evaluation of some aspects in supervised cell type identification

for single-cell RNA-seq: classifier, feature selection, and reference construction. Genome Biol 2021; **22**(1): 1-23.

McLean CY, Bristor D, Hiller M, et al. GREAT improves functional interpretation of cis-regulatory regions. Nat Biotechnol 2010; **28**(5): 495-501.

Nehma G and Tiwari M. Leveraging KANs for enhanced deep koopman operator discovery. arXiv preprint arXiv:2406.02875 2024.

Sherman BT, Hao M, Qiu J, et al. DAVID: a web server for functional enrichment analysis and functional annotation of gene lists (2021 update). Nucleic Acids Res 2022; **50**(W1): W216-W221.

Slowikowski K, Hu X and Raychaudhuri S. SNPsea: An algorithm to identify cell types, tissues and pathways affected by risk loci. Bioinformatics 2014; **30**(17): 2496-2497.

Tang S, Cui X, Wang R, et al. scCASE: accurate and interpretable enhancement for single-cell chromatin accessibility sequencing data. Nat Commun 2024; **15**(1): 1629.

Wolf FA, Angerer P and Theis FJ. SCANPY: Large-scale single-cell gene expression data analysis. Genome Biol 2018; **19**(1): 1-5.

Zeng Y, Luo M, Shangguan N, et al. Deciphering cell types by integrating scATAC-seq data with genome sequences. Nat Comput Sci 2024; **4**(4): 285-298.
